# Accurate graph classification via two-staged contrastive curriculum learning

**DOI:** 10.1371/journal.pone.0296171

**Published:** 2024-01-03

**Authors:** Sooyeon Shim, Junghun Kim, Kahyun Park, U. Kang

**Affiliations:** Department of Computer Science and Engineering, Seoul National University, Seoul, Republic of Korea; Shanghai Maritime University, CHINA

## Abstract

Given a graph dataset, how can we generate meaningful graph representations that maximize classification accuracy? Learning representative graph embeddings is important for solving various real-world graph-based tasks. Graph contrastive learning aims to learn representations of graphs by capturing the relationship between the original graph and the augmented graph. However, previous contrastive learning methods neither capture semantic information within graphs nor consider both nodes and graphs while learning graph embeddings. We propose TAG (Two-staged contrAstive curriculum learning for Graphs), a two-staged contrastive learning method for graph classification. TAG learns graph representations in two levels: node-level and graph level, by exploiting six degree-based model-agnostic augmentation algorithms. Experiments show that TAG outperforms both unsupervised and supervised methods in classification accuracy, achieving up to 4.08% points and 4.76% points higher than the second-best unsupervised and supervised methods on average, respectively.

## Introduction

*How can we generate graph representations for accurate graph classification?* Graph neural network (GNN) has drawn the attention of researchers since it is applicable to real-world graph-structured data including social networks, molecular graphs, etc. Various GNNs have been proposed to solve graph classification [1–7].

A main challenge of accurate graph classification is to learn graph embeddings that reflect the crucial information within graphs. Contrastive learning has been widely used to address the issue and achieved superior performance on the graph classification task. Graph contrastive learning produces the representations of graphs based on the similarity between graphs. The learning algorithm can be used in both settings: unsupervised [8–14] and supervised settings [15, 16].

Recent graph contrastive learning methods utilize data augmentation to ensure the similarity of the original graph and the newly generated graph. Random-based augmentations are used to generate graphs in [9, 10, 13], but information loss is inevitable in those methods. Graph contrastive learning methods with carefully designed augmentations [8, 11, 12, 14] preserve more graph semantics compared to those with random-based ones; however, these methods increase the complexity of their models. Furthermore, none of the previous approaches optimize node embeddings which are the basis of graph embeddings.

**Fig 1:**
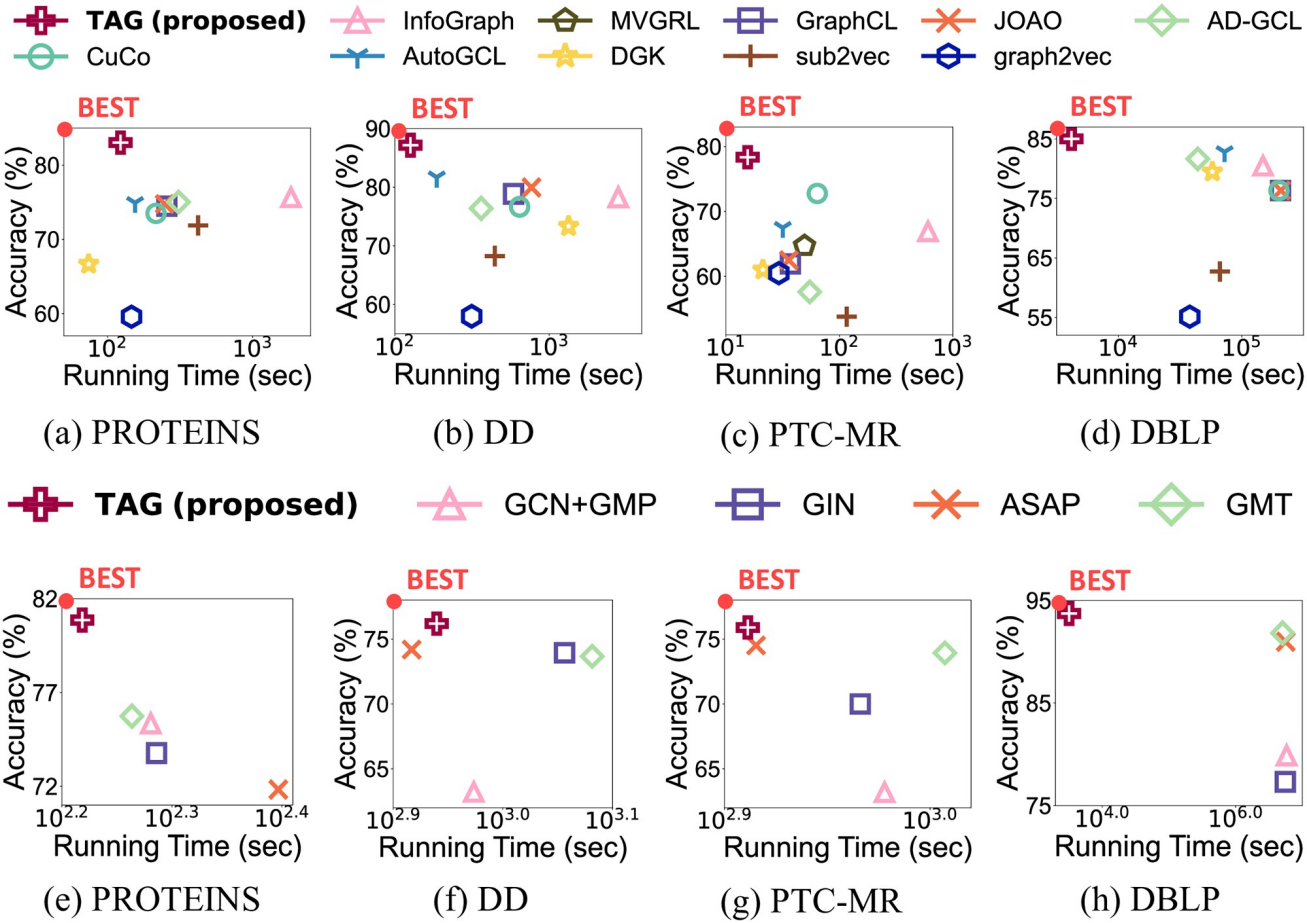
Overall performance of TAG in unsupervised and supervised graph classification. (a-d) are the performance in unsupervised setting, and (e-h) are that in supervised one. Note that TAG shows the highest classification accuracy with the shortest running time in both settings.

In this paper, we propose TAG (Two-staged contrAstive curriculum learning for Graphs), an accurate graph contrastive learning approach that can be applied to both supervised and unsupervised graph classification. We design six model-agnostic augmentation algorithms that preserve the semantic information of graphs. Three algorithms change the features of nodes, and the other three modify the structure of graphs based on degree centrality. We then conduct graph contrastive learning in two levels: node-level and graph-level. Node-level contrastive learning learns node embeddings based on the relationship between nodes. Graph-level contrastive learning learns the embeddings of graphs based on node embeddings. The embeddings of all nodes within a graph are aggregated to generate a graph embedding. Thus, the relationships of both nodes and graphs are reflected in the graph representations. Furthermore, TAG exploits a curriculum learning strategy to enhance performance. Fig 1 shows the overall performance of TAG; note that TAG outperforms the competitors in both unsupervised and supervised settings.

Our main contributions are summarized as follows:

- **Data augmentation.** We propose six model-agnostic augmentation algorithms for graphs. Every augmentation method considers node centrality to preserve semantic information of original graphs.
- **Method.** We propose TAG, a two-staged contrastive curriculum learning method for accurate graph classification. The two-staged approach embeds the relational information of both nodes and graphs into the graph representations.
- **Experiments.** We perform experiments on seven benchmark datasets in supervised and unsupervised settings, achieving the best performance.

Table 1 describes the symbols used in this paper. The code is available at https://github.com/snudatalab/TAG.

**Table 1:** Description of symbols.

Symbol	Description
D	a set of graphs for training
*G*_*i*_	*i*-th graph in a set D
*G*_*f*,*i*_	feature-modified graph originated from *G*_*i*_
*G*_*s*,*i*_	structure-modified graph originated from *G*_*i*_
*G*_*s*,*i*′_	structure-modified graph originated from *G*_*i*′_ for *i* ≠ *i*′
*v*_*j*_	*j*-th node in a graph *G*_*i*_
*u*_*j*_	*j*-th node in a graph *G*_*f*,*i*_

## Related works

### Node-level graph contrastive learning

Node-level graph contrastive learning methods are designed to handle node classification task by capturing the relationship between nodes. DGI [17] is the first work that applies the concept of contrastive learning to the graph domain. JGCL [18] combines supervised setting, semi-supervised setting, and unsupervised setting to learn the optimal node representations. GMI [19] defines the concept of graph mutual information (GMI) and aims to maximize the mutual information in terms of node features and topology of graphs. GCC [20] learns transferable structural representation across various networks to guide the pre-training of graph neural networks. GRACE [21] jointly considers both topology and node attribute levels for corruption to generate graph views and maximizes the agreement in the views at the node level. Zhu et al. [22] propose GCA which removes unimportant edges by giving them large removal probabilities on the topology level and adds more noise to unimportant feature dimensions on the node attribute level for adaptive augmentation. BGRL [23] is a scalable method with two encoders that learns by predicting alternative augmentations of the input. Graph Barlow Twins (G-BT) [24] is a model that replaces negative samples with a cross-correlation-based loss function and does not introduce asymmetry in the network. black However, those previous approaches for node-level graph contrastive learning address only the node classification problem, making them unsuitable for graph classification problem.

### Graph-level graph contrastive learning

Graph-level graph contrastive learning aims to obtain graph representations to solve graph classification task. Previous graph-level contrastive learning methods are divided into two types: model-specific and model-agnostic ones. Model-agnostic approaches use augmentation algorithms which do not engage in the training process. GraphCL [10] brings the contrastive learning method for images to the graph domain. CuCo [13] extends GraphCL by applying curriculum learning to properly learn from the negative samples. MVGRL [9] learns graph-level representations by contrasting encodings from first-order neighbors and graph diffusion. These methods use random-based graph augmentations that cannot preserve the core information of graphs well. We propose a graph contrastive learning method along with degree-based augmentations to address the issue.

Model-specific augmentation approaches directly participate in the training process. InfoGraph [8] learns graph representations by contrasting them with patch-level representations obtained from the training process. You et al. [11] propose JOAO which changes the simple augmentations to be learnable. AD-GCL [12] adopts the structure of an adversarial attack to obtain graph representations. AutoGCL [14] generates new graphs by changing the softmax function into the Gumbel-Softmax function. black However, those approaches for graph-level graph contrastive learning are more complex than model-agnostic methods, significantly increasing the training time. Therefore, we propose a contrastive learning method with simple augmentations for computational efficiency.

### Graph augmentation

Data augmentation has garnered significant attention recently, due to its successful application to many domains including image classification [25], natural language processing (NLP) [26], human activity recognition (HAR) [27, 28], and cognitive engagement classification [29]. Among them, graph augmentation methods are actively studied for improving the performance of graph contrastive learning.

Graph augmentation algorithms are divided into two types: model-specific and model-agnostic augmentation. Model-specific augmentation algorithms are restricted to a certain model. black Thus, those augmentation methods are not easy to be directly used in graph contrastive learning.

Model-agnostic graph augmentations are applied to any graph neural network. You et al. [10] suggest DropNode and ChangeAttr for graph contrastive learning. DropNode discards randomly selected nodes with their connections and ChangeAttr converts features of randomly selected nodes into random values. DropEdge [30] changes graph topology by removing a certain ratio of edges. GraphCrop [31] selects a subgraph from a graph through a random walk. Wang et al. [32] introduce NodeAug which contains three different augmentations: ReplaceAttr, RemoveEdge, and AddEdge. ReplaceAttr substitutes the feature of a chosen node with the average of its neighboring nodes’ features. RemoveEdge discards edges based on the importance score of edges. AddEdge attaches new edges to a central node which is designated based on the importance score for nodes. Motif-similarity [33] adds and deletes edges from motifs that are frequent in a particular graph. Yoo et al. [34] proposes NodeSam and SubMix. NodeSam performs split and merge operations on nodes. SubMix replaces a subgraph of a graph with another subgraph cut off from another graph. black SFA [35] proposes a spectral feature argumentation for contrastive learning on graphs.

However, previous model-agnostic augmentation algorithms [10, 31–34] change nodes or edges that are randomly selected, which easily overlook the semantic information of the original graphs. Another limitation is that previous approaches change only node attributes [35] or graph structures [30, 33], restricting the diversity of augmented examples. On the other hand, TAG changes both node attributes and graph structures based on the degree centrality to preserve crucial information of graphs.

## Preliminary on graph contrastive learning

In this section, we describe the preliminary of our work. Contrastive learning aims to learn embeddings by capturing the relational information between instances. For each instance, positive and negative samples need to be defined to maximize the similarity between a given instance and a positive sample compared to negative samples. Graph contrastive learning operates on graph-structured data. Recent works utilize data augmentation to generate positive samples. Previous graph contrastive learning methods are divided into two categories: node-level and graph-level contrastive learning.

Node-level graph contrastive learning methods obtain node embeddings of a graph. Given a graph, previous approaches augment the given graph and contrast nodes of the given graph and the augmented graph. A pair of nodes from two graphs at the same position is defined as positive samples and all other nodes except for positive samples are defined as negative samples. The model then learns the similarity of a positive pair against a negative pair. Graph-level graph contrastive learning methods learn graph embeddings by contrasting the graphs. Previous approaches set two augmented graphs with the same origin as positive samples and all other graphs in the training set except for the original graph as negative samples. Graph-level contrastive learning models then capture the similarity between a positive pair of graphs compared to a negative pair.

Despite the decent performance of graph contrastive learning, there is still a room for improvement. First, the relationship between node and graph embeddings has not been studied. Even though graph embeddings are obtained based on node embeddings, previous graph contrastive learning methods do not consider node embeddings. Second, most augmentation algorithms for contrastive learning randomly select nodes or edges to be modified. Since node feature and graph topology are the most essential components of graph-structured data, augmenting graphs while preserving crucial information within the pivotal components is important. However, previous methods rely on random-based augmentation algorithms which inevitably involve information loss. Finally, the influence of both positive and negative samples has not been studied. Previous methods focus on either positive or negative samples. To improve the performance of graph contrastive learning, well-defining both positive and negative samples is important. In this work, we propose TAG which addresses the three issues.

## Proposed method

We propose TAG, a two-staged contrastive curriculum learning framework for graphs. The main challenges and our approaches are as follows:

1. **How can we generate graph representations in both unsupervised and supervised settings?** We propose a two-staged graph contrastive curriculum learning method that is applied to both settings through two types of loss functions.
2. **How can we design augmentations for contrastive learning to preserve the semantics well?** We propose six data augmentation algorithms for graph contrastive learning. The augmentation algorithms consider degree centrality to minimize information loss.
3. **How can we determine the order of feeding the negative examples in contrastive learning?** We exploit curriculum learning to determine the order of negative samples and maximize the performance of the model.

**Fig 2:**
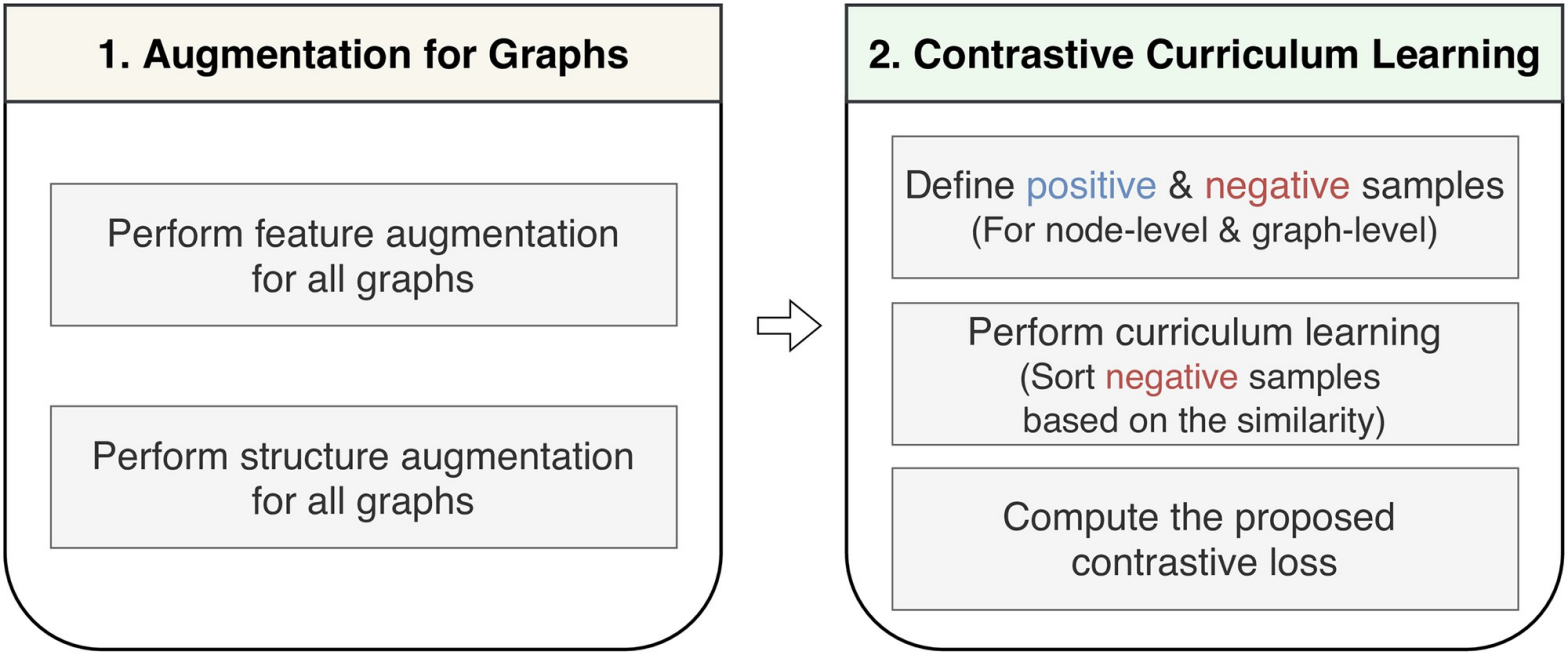
Overview of the proposed method. TAG first augments all graphs in a training set D, and then performs node-level and graph-level contrastive curriculum learning. For contrastive learning, TAG defines positive and negative samples, and computes the similarity between them. The proposed method learns negative samples from easy to hard ones which is determined based on the similarity.

**Fig 3:**
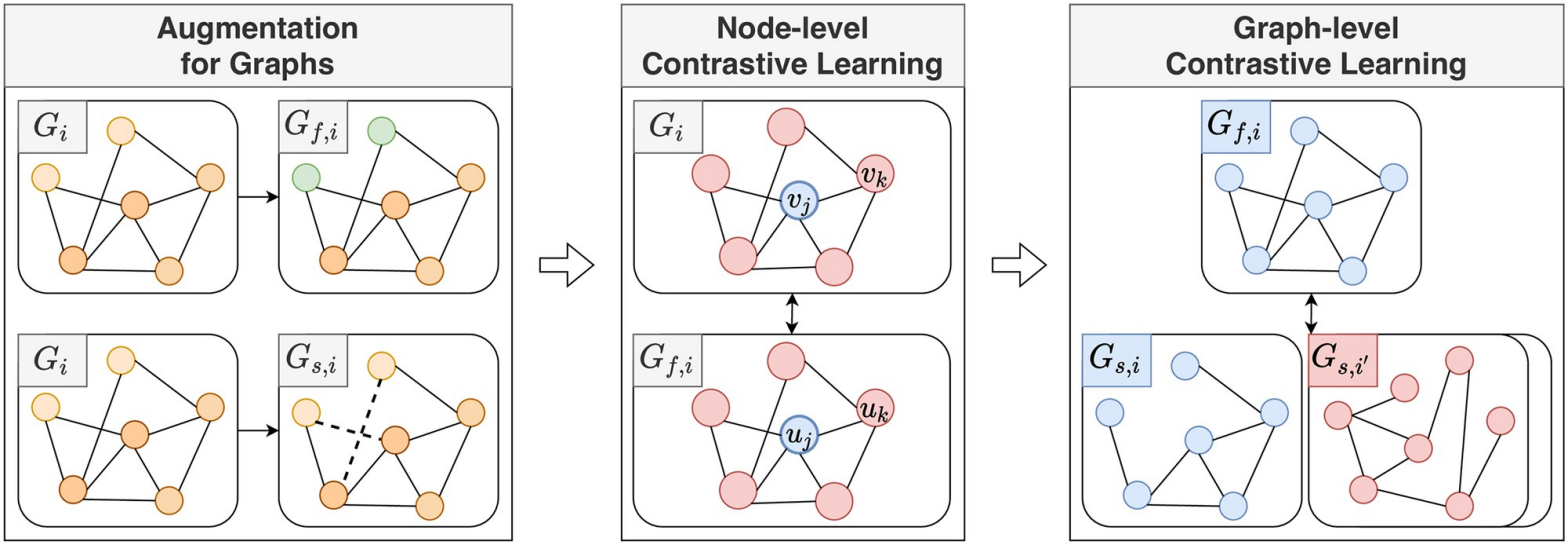
Example of the overall process of the proposed method. TAG performs node-level and graph-level contrastive learning on the feature-augmented graph *G*_*f*,*i*_ and the structure-augmented graph *G*_*s*,*i*_ obtained from the original graph *G*_*i*_. In the contrastive learning steps, nodes and graphs colored with blue are positive samples, and those colored with red are negative ones.

The overall process of TAG is illustrated in Figs 2 and 3. Fig 2 explains how the proposed method learns a training set. Fig 3 illustrates the details of performing augmentation and contrastive learning. Given a graph dataset, we first augment graphs, and then perform contrastive curriculum learning in two levels: nodes and graphs.

### Data augmentation

Our goal is to design data augmentation algorithms that minimize the information loss of graphs. Data augmentation is used to ensure the similarity between samples in contrastive learning. The most important challenge of augmentation is preserving the semantics, or keeping crucial information in determining graph labels. If the semantics are not preserved well in the process of augmentation, the original graph and the augmented graph would have different labels, resulting in increased dissimilarity. Therefore, we propose six model-agnostic graph augmentation algorithms based on degree centrality to minimize information loss. Our idea is to change low-degree nodes to minimize the loss of semantics.

We categorize the six augmentation methods into two types: feature and structure modification. Feature modification algorithms generate new graphs by changing only the node feature. On the other hand, structure modification algorithms change the graph structure. We propose three algorithms for each type. The three algorithms designed for feature augmentation are listed as follows:

1. **Edit feature.** Randomly change the features of *n* nodes with the lowest degrees.
2. **Mix feature.** Mix the features of two selected nodes and then substitute the mixed features for the features of nodes with lower degrees. Repeat this process *n* times.
3. **Add noise.** Add noise to the features of selected nodes. *n* nodes with the lowest degrees are selected to be modified.

The algorithms for structure augmentation are as follows:

1. **Delete node.** Discard *n* nodes with the lowest degrees along with their connections.
2. **Delete edge.** Select *m* edges from nodes with the lowest degrees. Remove the selected edges.
3. **Cut subgraph.** Select a subgraph with high-degree nodes.

*n* and *m* denote the number of nodes and edges to be modified, respectively.*n* and *m* are decided according to the augmentation ratio which is given as a hyperparameter. All algorithms consider degree centrality to keep semantic information.

**Algorithm 1** TAG (Two-staged Contrastive Curriculum Learning for Graphs)

**Input:** training set $D={\left\{G_{i}\right\}}_{i=1}^{N}$ of graphs, graph neural network *f* with parameters *θ*, and number *T* of training epochs

**Output:** the trained graph neural network *f*

1: **for** $G_{i}\in D$ **do**

2:  $A_{f}\leftarrow$ select a feature modification algorithm at random

3: $A_{s}\leftarrow$ select a structure modification algorithm at random

4:  *G*_*f*,*i*_, *G*_*s*,*i*_← augment a graph *G*_*i*_ with $A_{f},A_{s}$

5: **end for**

6: **for** *t* ← 1 to *T* **do**

7:  **for** *i* ← 1 to *N* **do**

8:   *l*_n_(*i*) ← *ContrastNode*(*G*_*i*_, *G*_*f*,*i*_, *f*)     ⊳ Algorithm 2

9:   $l_{g}\left(i\right)\leftarrow \text{ContrastGraph}\left(G_{f,i},{\left\{G_{s,i}\right\}}_{i=1}^{N},f\right)$     ⊳ Algorithm 3

10:  **end for**

11:  $L\leftarrow -\left(1/N\right)\Sigma_{i\in D}\left(l_{n}\left(i\right)+l_{g}\left(i\right)\right)$     ⊳ Eq 3

12:  *θ* ← update the parameters to minimize L

13: **end for**

### Two-staged contrastive learning

We propose a graph contrastive learning model for accurate graph classification utilizing all the proposed augmentation algorithms. Graph contrastive learning is a self-supervised approach that allows a model to learn the representations of graphs without labels by teaching the model which graph instances are similar or different. We use the data augmentation algorithms proposed in the Data augmentation section to generate similar graphs. Considering the fact that graph embeddings are obtained based on node embeddings, learning the representative embeddings from both nodes and graphs is important. We propose TAG which conducts graph contrastive learning on two stages: node-level and graph-level.

Algorithm 1 shows the overall training process of TAG. Given a training set D of graphs, we first augment graphs in D before training, and then perform two-staged contrastive curriculum learning. Node-level contrastive curriculum learning captures the relational information between nodes in a graph *G*_*i*_ and a feature-modified graph *G*_*f*,*i*_ (line 8 in Algorithm 1). Graph-level contrastive learning extracts representative graph embeddings by maximizing the similarity between graphs *G*_*f*,*i*_ and *G*_*s*,*i*_ with the same origin (line 9 in Algorithm 1). A graph neural network is trained by minimizing the proposed two-staged contrastive loss (line 12 in Algorithm 1).

In the following, we first explain the two-staged approach of TAG in detail. Then, we describe how to apply TAG for supervised graph classification and how to exploit curriculum learning for determining the order of negative samples.

**Algorithm 2** ContrastNode in TAG

**Input:** original graph $G_{i}=\left(V_{i},E_{i},X_{i}\right)$, feature-augmented graph $G_{f,i}=\left(V_{f,i},E_{f,i},X_{f,i}\right)$, and graph neural network *f* with parameters *θ*

**Output:** node-level contrastive loss *l*_n_(*i*) for a graph *G*_*i*_

1: **for** $j\in V_{i}$ **do**

2:  (*v*_*j*_, *u*_*j*_) ← select a positive pair of nodes from $V_{i},V_{f,i}$

3:  **for** $k\in V_{i}$ **do**

4:   **if** *k* ≠ *j* **then**

5:   *v*_*k*_, *u*_*k*_ ← select negative nodes from $V_{i},V_{f,i}$

6:    **x**_*j*_, **x**_*k*_, **x**_*f*,*j*_, **x**_*f*,*k*_ ← get feature vectors of nodes *v*_*j*_, *v*_*k*_, *u*_*j*_, *u*_*k*_

7:    **v**_*j*_, **v**_*k*_, **u**_*j*_, **u**_*k*_ ← *f*(**x**_*j*_, *θ*), *f*(**x**_*k*_, *θ*), *f*(**x**_*f*,*j*_, *θ*), *f*(**x**_*f*,*k*_, *θ*)

8:    $S\left(v_{j},v_{k}\right)\leftarrow |v_{j}\cdot v_{k}|/|v_{j}\left|\right|v_{k}|$

9:    $S\left(v_{j},u_{k}\right)\leftarrow |v_{j}\cdot u_{k}|/|v_{j}\left|\right|u_{k}|$

10:   **end if**

11:  **end for**

12:  Sort negative nodes according to S in the ascending order

13: **end for**

14: Compute *l*_n_(*i*)     ⊳ Eq 1

#### Node-level contrastive learning

The objective of the node-level contrastive learning in TAG is to learn meaningful node representations by embedding the nodes into a latent space where positive pairs of nodes are more closely located than negative ones. Positive pairs (*v*_*j*_, *u*_*j*_) of nodes are obtained by selecting a node *v*_*j*_ from an original graph *G*_*i*_, and a node *u*_*j*_ from a feature-augmented graph *G*_*f*,*i*_ with the same position. We utilize all of the proposed augmentations by randomly selecting an augmentation algorithm for a graph from the proposed algorithms.

**Fig 4:**
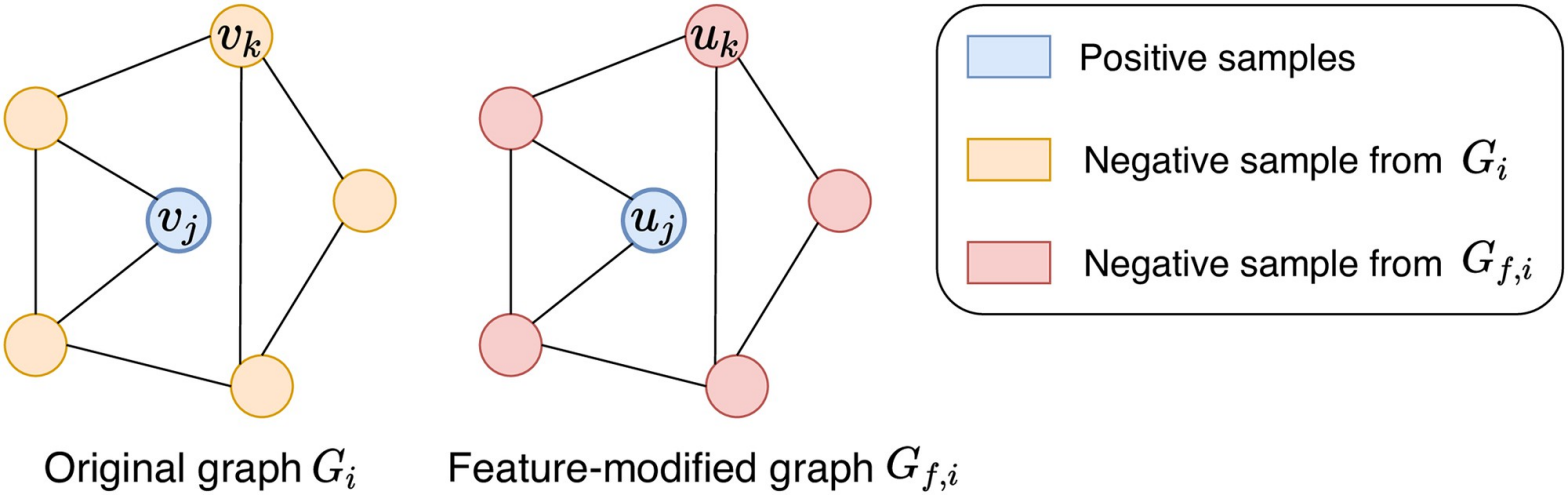
Positive and negative samples of the node-level contrastive learning. Nodes *v*_*j*_ and *v*_*k*_ are selected from the original graph *G*_*i*_ while nodes *u*_*j*_ and *u*_*k*_ are sampled from a feature-augmented graph *G*_*f*,*i*_ at the same position.

There are two types of negative node pairs: 1) pairs (*v*_*j*_, *v*_*k*_) of nodes both sampled from the original graph *G*_*i*_, and 2) pairs (*v*_*j*_, *u*_*k*_) of nodes sampled from *G*_*i*_ and *G*_*f*,*i*_, respectively. All nodes in *G*_*i*_ which are not selected for the positive pairs are used to generate the negative samples *v*_*k*_. Similarly, every node *u*_*k*_ from *G*_*f*,*i*_ except for the selected positive node *u*_*j*_ is treated as a negative sample. The process of sampling positive and negative pairs of nodes for the node-level contrastive learning is illustrated in Fig 4.

The node-level contrastive loss *l*_n_ is defined as follows:

$$\begin{array}{c} l_{n}=\sum_{j=1}^{K}\text{log}\frac{\text{exp}(\text{sim}\left(v_{j},u_{j}\right)/\tau )}{\sum_{k\neq j}^{K}\text{exp}\left(\text{sim}\left(v_{j},v_{k}\right)/\tau \right)+\sum_{k\neq j}^{K}\text{exp}\left(\text{sim}\left(v_{j},u_{k}\right)/\tau \right)} \end{array}$$ (1)

where sim(⋅) denotes the cosine similarity function, *τ* is the temperature parameter, and *K* is the number of nodes in a graph. Vectors **v**_*j*_ and **u**_*j*_ are the hidden representations of nodes *v*_*j*_ and *u*_*j*_, respectively. Algorithm 1 shows the process of calculating node-level contrastive loss. We exploit curriculum learning and compute the loss with reordered negative samples whose ordering is determined in line 12 of Algorithm 1. We feed negative samples from easy to hard ones where the difficulty of a negative sample is defined as the cosine similarity of the sample and its paired positive sample.

**Algorithm 3** ContrastGraph in TAG

**Input:** feature-augmented graph *G*_*f*,*i*_, structure-augmented graphs ${\left\{G_{s,i}\right\}}_{i=1}^{N}$, and graph neural network *f* with parameters *θ*

**Output:** raph-level contrastive loss *l*_g_(*i*) for a graph *G*_*i*_

1: (*G*_*f*,*i*_, *G*_*s*,*i*_) ← select a positive pair of graphs

2:  *i*′ ← 1 to *N* **do**

3:   **if** *i*′ ≠ *i* **then**

4:    *G*_*s*,*i*′_ ← select a negative graph

5:    **z**_*f*,*i*_, **z**_*s*,*i*′_ ← average node embeddings within *G*_*f*,*i*_, *G*_*s*,*i*′_

6:    $S\left(G_{f,i},G_{s,i^{\prime }}\right)\leftarrow |z_{f,i}\cdot z_{s,i^{\prime }}|/|z_{f,i}\left|\right|z_{s,i^{\prime }}|$

7:   **end if**

8:  **end for**

9: Sort negative graphs according to S in the ascending order

10: Compute *l*_g_(*i*)    ⊳ Eq 2

#### Graph-level contrastive learning

Graph-level contrastive learning in TAG aims to obtain representative graph embeddings. Graph embeddings are learned by collecting all node embeddings within a graph with the average function. As with node-level contrastive learning, positive and negative samples are defined using augmentation in graph-level contrastive learning.

**Fig 5:**
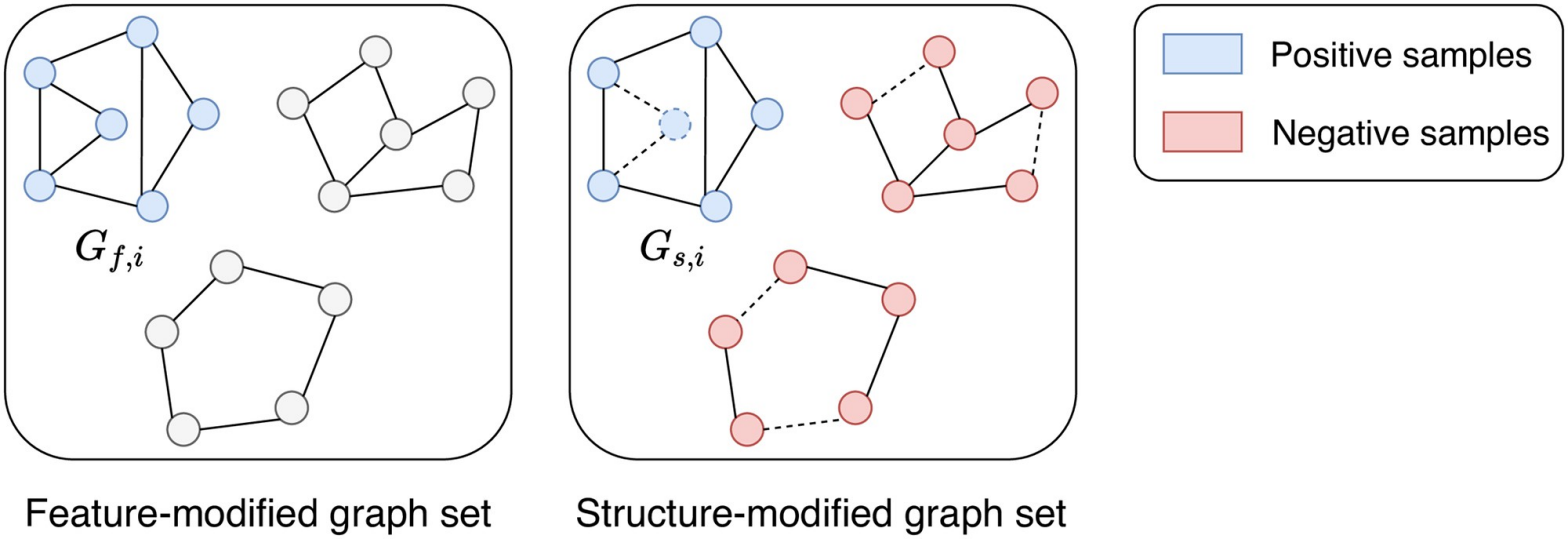
Illustration of positive and negative samples for graph-level contrastive learning. (*G*_*f*,*i*_, *G*_*s*,*i*_) is a positive pair originated from a graph *G*_*i*_, and (*G*_*f*,*i*_, *G*_*s*,*i*′_) for *i* ≠ *i*′ are negative pairs.

A positive pair (*G*_*f*,*i*_, *G*_*s*,*i*_) of graphs contains a feature-modified graph *G*_*f*,*i*_ and a structure-modified graph *G*_*s*,*i*_ of a graph *G*_*i*_. Feature modification and structure modification algorithms are randomly chosen from the proposed augmentation algorithms. Negative pairs are (*G*_*f*,*i*_, *G*_*s*,*i*′_) where *G*_*i*′_ is a different graph from *G*_*i*_. Fig 5 explains positive and negative samples designed for graph-level contrastive learning.

The graph-level contrastive loss *l*_g_ is written as below:

$$\begin{array}{c} l_{g}=\text{log}\frac{\text{exp}(\text{sim}\left(z_{f,i},z_{s,i}\right)/\tau )}{\sum_{i^{\prime }\neq i}^{N}\text{exp}\left(\text{sim}\left(z_{f,i},z_{s,i^{\prime }}\right)/\tau \right)} \end{array}$$ (2)

where **z**_⋅,*i*_ is a representation of graph *G*_⋅,*i*_ and *N* is the number of graphs for training. Algorithm 3 describes the process of calculating graph-level contrastive loss where graph representations are obtained based on node representations in line 5. We reorder the negative samples in line 9 of Algorithm 3 to maximize the performance of TAG by exploiting curriculum learning. TAG trains the samples gradually from easy to hard ones where a negative pair of graphs with low similarity is regarded as an easy sample.

The final loss function L for TAG jointly uses the node-level and graph-level contrastive losses. Given a set D of graphs for training,

$$\begin{array}{c} L=-\frac{1}{\left|D\right|}\sum_{i\in D}\left(l_{n}\left(i\right)+l_{g}\left(i\right)\right) \end{array}$$ (3)

where *l*_n_(*i*) and *l*_g_(*i*) are node- and graph-level losses for a graph *G*_*i*_, respectively.

#### Supervised contrastive learning

To further improve the performance of TAG, we design the proposed method to operate in the supervised setting as well. In supervised graph classification, the labels of graphs are available while training. To exploit the information of the given labels, we use the typical cross-entropy loss *l*_ce_(⋅). Specifically, the loss $l_{\text{ce}}\left(y_{i},{\hat{y}}_{i}\right)$ between the one-hot encoded label **y**_*i*_ and the prediction probability ${\hat{y}}_{i}$ of a graph *G*_*i*_ is computed as follows:

$$\begin{array}{c} l_{\text{ce}}\left(y_{i},{\hat{y}}_{i}\right)=\sum_{c=1}^{C}y_{i}\left(c\right)\text{log}\;{\hat{y}}_{i}\left(c\right) \end{array}$$ (4)

where *C* is the number of classes, *y*_*i*_(*c*) is *c*-th element of **y**_*i*_, and ${\hat{y}}_{i}\left(c\right)$ is the prediction probability of a graph *G*_*i*_ to class *c*. Node and graph representation vectors in Eqs 1 and 2 are learned using a graph neural network. For supervised graph classification, we attach a fully-connected layer to the final layer of the graph neural network to construct TAG as an end-to-end model. The probability vector ${\hat{y}}_{i}$ is obtained through the softmax function after a fully-connected layer.

To fully exploit both the result of the two-staged contrastive learning and the information of given labels while training, we minimize the supervised loss $l_{\text{ce}}\left(y_{i},{\hat{y}}_{i}\right)$ and the two-staged contrastive loss L simultaneously. Thus, the loss $L_{\text{sup}}$ for supervised learning is computed by adding the cross-entropy loss to the loss in Eq (3):

$$\begin{array}{c} L_{\text{sup}}=-\frac{1}{N}\sum_{i=1}^{N}\left(l_{n}\left(i\right)+l_{g}\left(i\right)+l_{\text{ce}}\left(y_{i},{\hat{y}}_{i}\right)\right) \end{array}$$ (5)

where *l*_n_(*i*) and *l*_g_(*i*) are node- and graph-level losses for a graph *G*_*i*_, respectively.*N* denotes the size of a set D.

### Curriculum learning

Curriculum learning imitates the learning process of humans who start learning from easier samples, and then learn more from harder samples. To further improve the performance of TAG, we reorder the samples for training by exploiting the curriculum learning strategy. A naive approach would define negative samples that are misclassified with high probability as hard samples. However, this is not directly applicable to the contrastive learning methods including TAG since the labels may not be given.

To determine the difficulty of samples regarding the two-staged contrastive loss, we utilize the similarity between positive and negative samples. The sample with a large loss is hard to learn because loss minimization is difficult. However, it is hard to use the loss as a difficulty measure since reordering should be done before loss calculation. Thus, we define the cosine similarity of nodes in a negative pair which affects the size of the loss as a difficulty score. If a negative sample is similar to a positive sample, the model struggles to find the difference between the samples causing a large loss. We feed negative samples with lower similarity first, and then move on to harder negative samples as training continues to facilitate effective training. Both node-level and graph-level contrastive learning train negative samples gradually from easy to hard ones.

## Experiments

We perform experiments to answer the following questions:

Q1. **Performance on Unsupervised Classification.** How fast and accurate is TAG compared to previous methods for unsupervised graph classification?
Q2. **Performance on Supervised Classification.** Does TAG show superior performance than other baselines in supervised graph classification task?
Q3. **Effectiveness of Proposed Augmentations.** Do the proposed augmentation algorithms improve the performance of TAG?
Q4. **Ablation Study.** Does each step of TAG contribute to the performance of the unsupervised graph classification task?

### Experimental settings

We introduce our experimental settings including datasets, competitors, and hyperparameters. All of our experiments are conducted on a single GPU machine with GeForce GTX 1080 Ti.

**Datasets.** We use seven benchmark datasets for graph classification task in our experiments, which are summarized in Table 2. MUTAG, PROTEINS, NCI1, NCI109, DD, and PTC-MR [36] are molecular datasets where the nodes stand for atoms and are labeled by the atom type, while edges are bonds between the atoms. DBLP [37] is a citation network dataset in the computer science field whose nodes represent scientific publications.

**Table 2:** Summarization of datasets.

Dataset	Graphs	Nodes	Edges	Features	Classes
**MUTAG** ^1^	188	3,371	3,721	7	2
**PROTEINS** ^1^	1,113	43,471	81,044	3	2
**NCI1** ^1^	4,110	122,747	132,753	37	2
**NCI109** ^1^	4,127	122,494	132,604	38	2
**DD** ^1^	1,178	334,925	843,046	89	2
**PTC-MR** ^1^	344	4,915	5,054	18	2
**DBLP** ^1^	19,456	203,954	764,512	41,325	2

^1^ https://chrsmrrs.github.io/datasets/

**Competitors.** We compare TAG in supervised and unsupervised settings. For the unsupervised setting, we compare TAG with ten previous approaches for unsupervised graph classification, including those for contrastive learning.

- **DGK [38]** learns latent representations of graphs by adopting the concept of the skip-gram model.
- **sub2vec [39]** is an unsupervised learning algorithm that captures two properties of subgraphs: neighborhood and structure.
- **graph2vec [40]** extends neural networks for document embedding to the graph domain, by viewing the graphs as documents.
- **InfoGraph [8]** generates graph representations by maximizing mutual information between graph-level and patch-level representations.
- **MVGRL [9]** learns graph representations by contrasting two diffusion matrices transformed from the adjacency matrix.
- **GraphCL [10]** brings image contrastive learning to graphs.
- **JOAO [11]** jointly optimizes augmentation selection together with the contrastive objectives.
- **AD-GCL [12]** uses an adversarial training strategy for edge-dropping augmentation of graphs.
- **CuCo [13]** adopts curriculum learning to graph contrastive learning for performance improvement.
- **AutoGCL [14]** uses node representations to predict the probability of selecting a certain augment operation.

We use support vector machine (SVM) and multi-layer perceptron (MLP) as base classifiers to evaluate the competitors and TAG in an unsupervised setting. We select an SVM classifier among various machine learning classifiers for a fair comparison since the competitors use SVM to evaluate their methods. To evaluate methods in deep learning as well as in machine learning, we exploit an MLP classifier.

In the supervised setting, we compare the accuracy of TAG with 4 baselines:

- **GCN+GMP [41]** uses the graph convolutional network (GCN) to learn the node representations, and the global mean pooling (GMP) is applied to obtain the graph representation.
- **GIN [5]** uses multi-layer perceptrons (MLP) to update node representations, and sums them up to generate the graph representation.
- **ASAP [6]** alternatively clusters nodes in a graph and gathers the representations of clusters to obtain graph representations.
- **GMT [7]** designs graph pooling layer based on multi-head attention.

We run 10-fold cross-validation to evaluate the competitors and TAG.

**Hyperparameters.** We use GCN [41] to learn node embeddings and apply the global mean pooling algorithm to generate a graph embedding. We set the augmentation ratio which decides the amount of data to be changed to 0.4. The ratio is the only hyperparameter for data augmentation of TAG. Thus, TAG does not suffer from hyperparameter optimization problems. We train each model using the Adam optimizer with a learning rate of 0.0001. We set the number of epochs to 5.

### Performance on unsupervised classification

We evaluate unsupervised graph classification accuracy and running time of TAG. The graph classification accuracy of TAG and previous unsupervised methods are described in Table 3. We adopt support vector machine (SVM) and multi-layer perceptron (MLP) as base classifiers for TAG and the baselines. Note that TAG achieves the best accuracy, giving 4.08% points and 2.14% points higher accuracy than the second-best competitors on average in SVM and MLP classifiers, respectively.

**Table 3:** Accuracy of graph classification in unsupervised setting. Bold and underlined text denote the best and the second-best accuracy, respectively. OOM and Avg. denote the out of memory error and average accuracy, respectively. Note that TAG shows the best classification accuracy.

Method	Unsupervised Setting (SVM)								Unsupervised Setting (MLP)							
	MUT.	PROT.	NCI1	N109	DD	PTC	DBLP	Avg.	MUT.	PROT.	NCI1	N109	DD	PTC	DBLP	Avg.
DGK [38]	85.67	66.67	65.47	65.86	73.35	61.03	79.49	71.08	66.46	67.40	54.18	53.02	62.31	49.10	75.25	61.10
sub2vec [39]	74.42	71.88	57.40	57.26	68.25	53.80	62.71	63.67	55.26	53.91	51.75	51.56	47.95	51.20	60.37	53.15
graph2vec [40]	68.57	59.57	54.04	52.41	57.98	60.50	55.12	58.31	64.94	56.79	54.36	52.17	55.34	55.84	54.69	56.30
InfoGraph [8]	85.12	75.65	**73.65**	72.52	78.27	66.92	80.48	76.09	71.78	69.28	60.27	**60.20**	73.07	57.61	76.49	66.96
MVGRL [9]	83.33	OOM	OOM	OOM	OOM	64.71	OOM	21.15	89.47	OOM	OOM	OOM	OOM	58.82	OOM	21.19
GraphCL [10]	86.67	74.48	66.89	67.26	78.86	61.93	76.33	73.20	75.06	69.37	60.10	59.49	71.73	60.50	75.28	67.36
JOAO [11]	88.25	74.76	66.84	67.17	79.96	62.50	76.34	73.69	75.53	69.28	60.17	59.71	73.18	63.19	75.21	68.04
AD-GCL [12]	88.82	75.03	71.85	71.46	76.39	57.61	81.65	74.69	81.76	62.14	58.09	59.56	61.08	55.97	77.51	65.16
CuCo [13]	87.31	73.50	64.33	63.83	76.66	72.78	76.34	73.54	71.23	66.59	60.46	58.89	**73.52**	57.25	73.46	65.91
AutoGCL [14]	89.42	74.93	71.43	**74.34**	81.69	67.50	82.82	77.45	84.21	70.54	**60.56**	59.98	72.03	68.03	79.06	70.63
**TAG**	**91.28**	**83.04**	73.47	72.38	**87.17**	**78.36**	**85.02**	**81.53**	**91.72**	**70.65**	60.53	58.63	72.52	**76.06**	**79.28**	**72.77**

**Fig 6:**
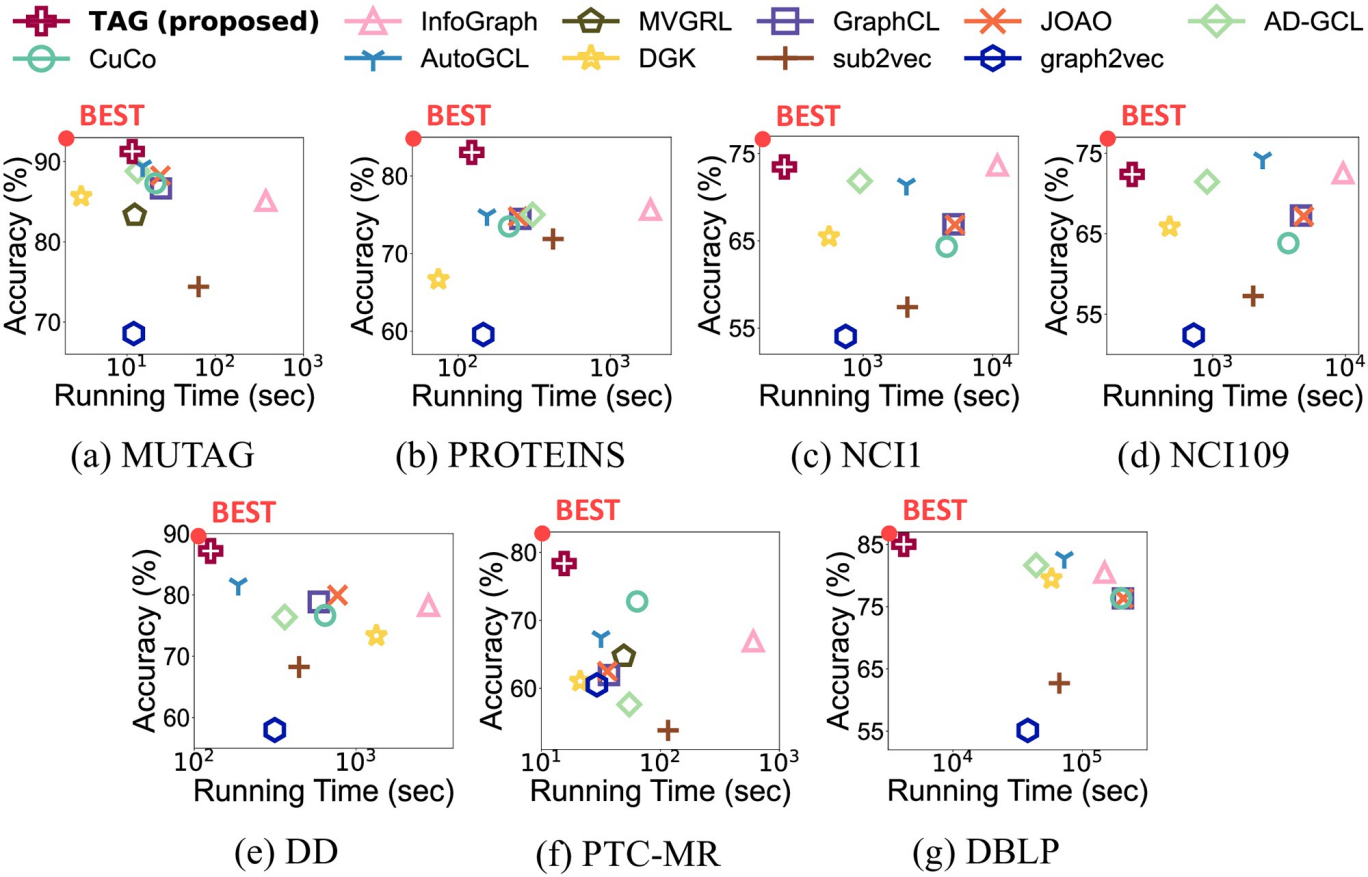
Overall performance of TAG and previous unsupervised graph classification methods with an SVM classifier. Note that TAG shows the highest classification accuracy with the shortest running time in most cases.

**Fig 7:**
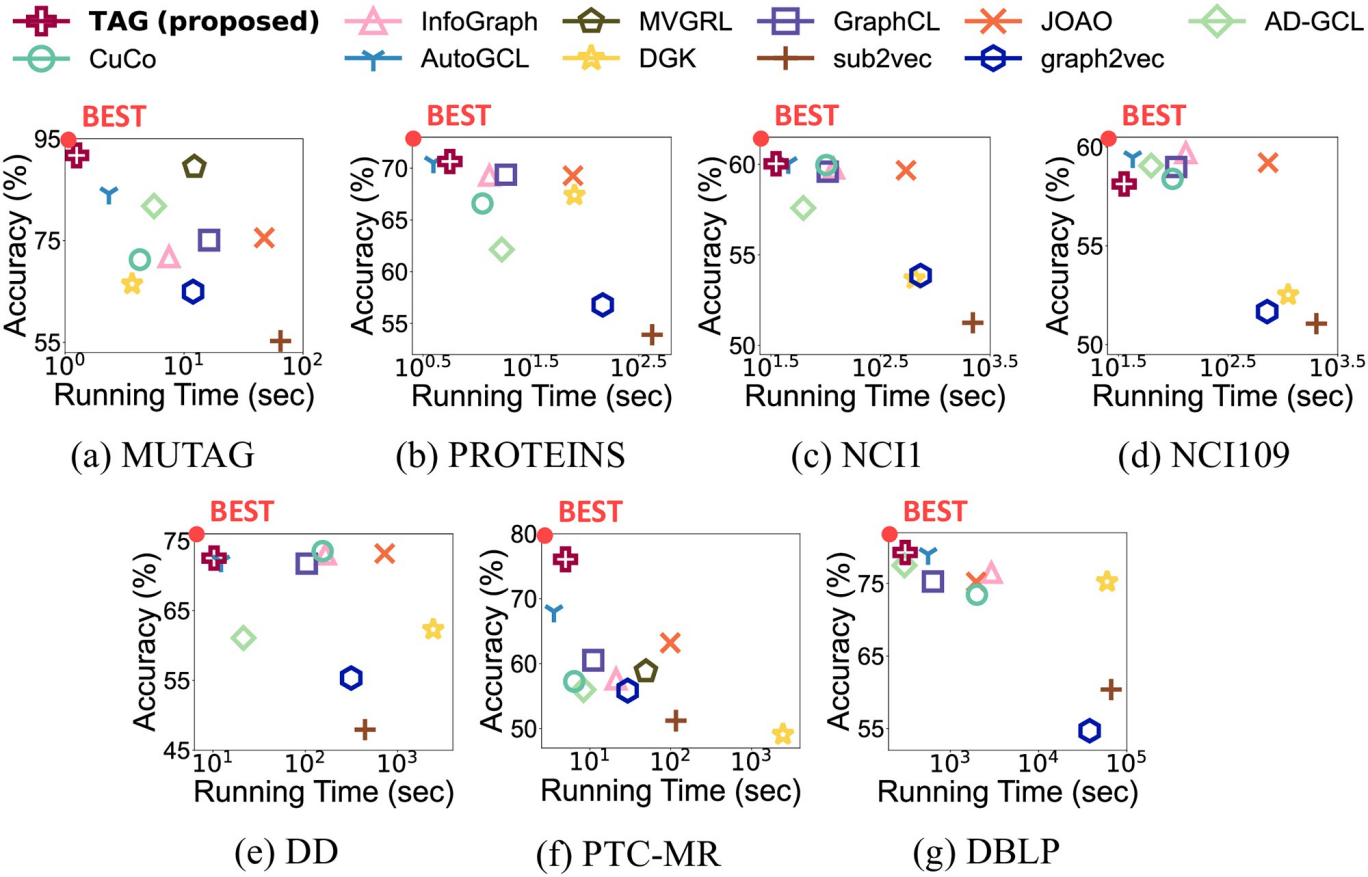
Overall performance of TAG and previous unsupervised graph classification methods with an MLP classifier. (a-g) show the accuracy and running time of each dataset. TAG outperforms the competitors in most cases.

The overall performance in the unsupervised setting of TAG with two classifiers including the running time is summarized in Figs 6 and 7. Fig 6 shows the results of TAG and previous approaches with an SVM classifier. Note that TAG shows the highest classification accuracy in most cases with the shortest running time. This shows that TAG effectively and efficiently finds the graph representations for unsupervised graph classification from large graphs. Fig 7 shows the accuracy and running time of TAG and the competitors measured with an MLP classifier. TAG outperforms the competitors for most datasets.

### Performance on supervised classification

TAG also operates in the supervised graph classification task in addition to the unsupervised one. We compare TAG with four baselines for supervised graph classification in Table 4. We use classification accuracy and running time as the evaluation metrics. Note that TAG gives the highest accuracy, with 4.76% points higher average accuracy than the second-best method. Specifically, TAG in the supervised setting achieves 4.50% points and 13.26% points higher average accuracy than that in the unsupervised setting in SVM and MLP classifiers, respectively.

**Table 4:** Accuracy of graph classification in supervised setting. Bold and underlined text denote the best and the second-best accuracy, respectively. Avg. denotes the average accuracy. Note that TAG shows the best accuracy.

Supervised Setting								
Method	MUT.	PROT.	NCI1	N109	DD	PTC	DBLP	Avg.
GCN+GMP [41]	82.35	73.56	63.21	63.19	62.00	75.35	79.88	71.36
GIN [5]	90.49	75.77	73.94	70.00	77.78	73.77	77.31	77.01
ASAP [6]	86.52	77.56	74.19	74.51	82.66	71.83	90.98	79.75
GMT [7]	89.07	79.73	73.67	73.93	84.95	75.74	91.82	81.27
**TAG**	**95.45**	**87.68**	**76.19**	**75.89**	**92.40**	**80.86**	**93.73**	**86.03**

**Fig 8:**
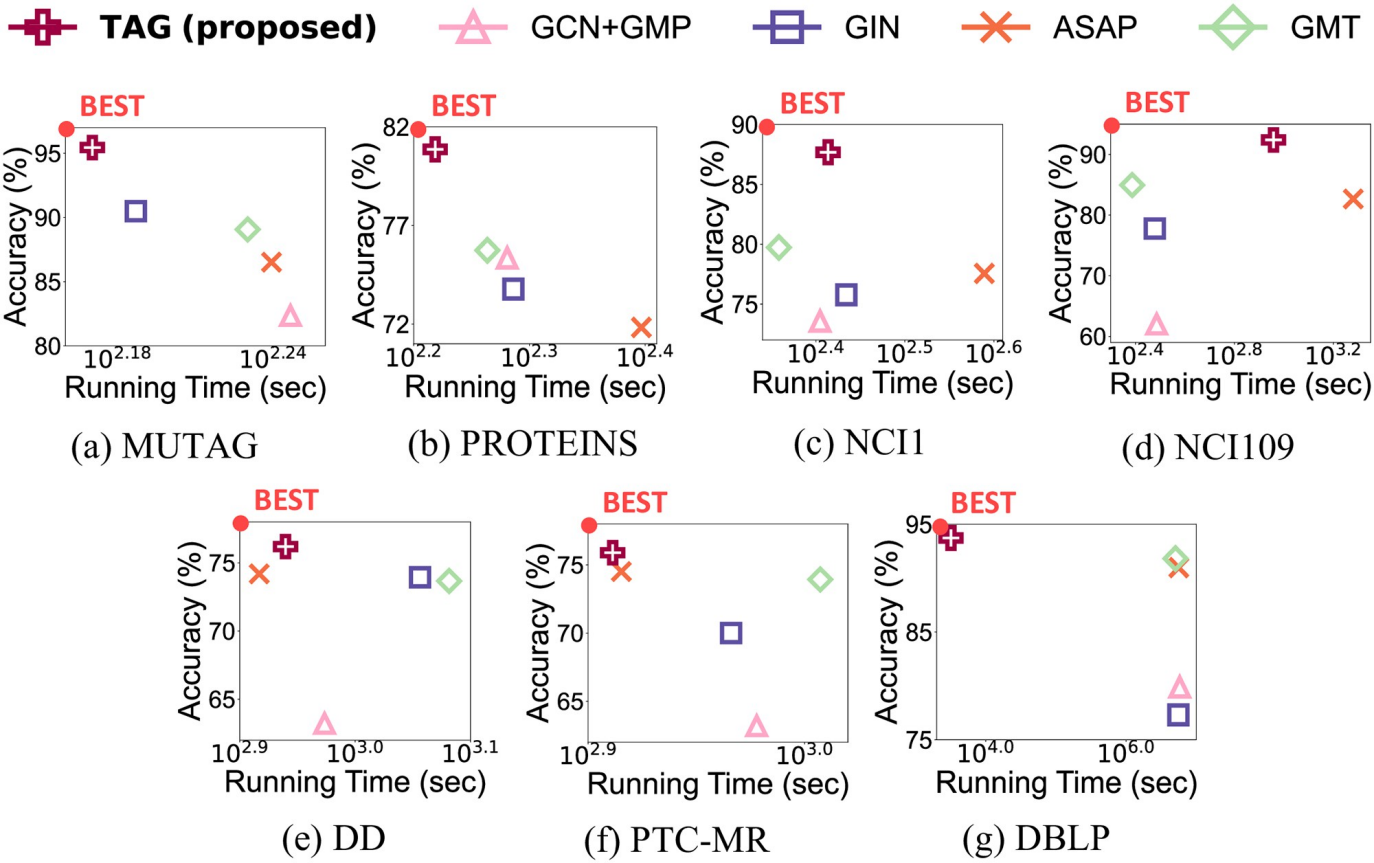
Overall performance of TAG with supervised graph classification methods. (a-g) show the performance in each dataset. Note that TAG shows the highest classification accuracy with the shortest running time for most datasets.

Fig 8 shows the classification accuracy and the running time of TAG and baselines in a supervised setting. Note that TAG gives the shortest running time with the highest accuracy in most of the cases. This shows that TAG efficiently learns meaningful graph representations not only for unsupervised graph classification, but also supervised one.

### Effectiveness of proposed augmentations

We compare the proposed augmentations of TAG with eight previous model-agnostic augmentation algorithms for graphs. ChangeAttr modifies features and the other methods change the structure of graphs. Recall that TAG performs graph contrastive learning in two levels: node-level and graph-level. For node-level, TAG needs feature-augmented graphs. For graph-level, TAG needs feature and structure augmentations. Thus, both augmentation algorithms are necessary for TAG. MVGRL [9], GraphCL [10], and CuCo [13] are previous methods that adopt model-agnostic graph augmentations. However, MVGRL causes out-of-memory errors for large-scale graph datasets. CuCo is more elaborate than GraphCL since it additionally performs curriculum learning. Therefore, we compare TAG with previous augmentation algorithms by applying them to CuCo.

Table 5 shows the classification results using different augmentations. The accuracy is measured with an SVM classifier. TAG outperforms the baselines in most cases. Specifically, TAG achieves 5.05% points higher average accuracy than the strongest baseline SubMix. Note that random-based augmentations DropNode, DropEdge, GraphCrop, and ChangeAttr degrade the performance of CuCo for all datasets. This proves that random-based augmentation methods have difficulty preserving the semantics. In contrast, TAG with the proposed augmentations help enhance the performance.

**Table 5:** Comparison of the augmentation methods. We report the best and the second-best accuracy with bold and underlined texts, respectively. Avg. denotes the average accuracy. Note that TAG presents the best accuracy among the models.

Method	MUT.	PROT.	NCI1	N109	DD	PTC	DBLP	Avg.
CuCo + DropNode [10]	87.31	73.50	64.36	63.80	76.75	60.52	76.33	71.80
CuCo + DropEdge [30]	88.86	72.61	63.72	63.41	77.50	64.81	76.33	72.46
CuCo + GraphCrop [31]	88.28	72.96	63.24	63.32	77.17	63.37	71.59	71.42
CuCo + ChangeAttr [10]	86.23	73.68	63.60	63.75	76.66	61.05	69.87	70.69
CuCo + NodeAug [32]	82.46	74.24	64.48	63.93	79.70	78.33	78.77	74.56
CuCo + Motif-Similarity [33]	90.00	70.68	66.64	63.92	78.79	77.50	77.19	74.96
CuCo + NodeSam [34]	89.11	76.74	64.23	64.69	82.05	**79.17**	78.98	76.42
CuCo + SubMix [34]	89.04	76.97	64.48	67.99	78.94	**79.17**	78.77	76.48
**TAG**	**91.28**	**83.04**	**73.47**	**72.38**	**87.17**	78.36	**85.02**	**81.53**

We also show the effectiveness of the degree-based node and edge selection of TAG for graph augmentation. We compare TAG with two different selection methods: TAG-random and TAG-reverse. TAG-random randomly selects the nodes or edges to be changed. TAG-reverse selects the nodes or edges from high to low degrees. Table 6 reports the classification accuracy of TAG and the baselines. We use SVM and MLP classifiers to measure the accuracy. Note that TAG outperforms the baselines in all datasets. Specifically, TAG achieves up to 4.36% points and 4.19% points higher average accuracy than the second-best baselines in SVM and MLP classifiers, respectively. This shows that the proposed augmentations of TAG considering the degree centrality effectively improves the graph classification accuracy.

**Table 6:** Effectiveness of degree centrality. TAG-random runs TAG by randomly selecting nodes or edges to be modified. TAG-reverse augments nodes or edges relevant to high degrees. Bold, underlined, and Avg. texts denote the best, the second-best, and the average accuracy, respectively.

Method	Unsupervised Setting (SVM)								Unsupervised Setting (MLP)							
	MUT.	PROT.	NCI1	N109	DD	PTC	DBLP	Avg.	MUT.	PROT.	NCI1	N109	DD	PTC	DBLP	Avg.
TAG-random	80.55	81.08	64.98	65.31	81.95	72.63	78.30	74.97	88.89	68.75	56.45	56.26	69.33	72.02	68.34	68.58
TAG-reverse	89.47	79.28	67.64	68.93	83.05	73.53	78.31	77.17	83.33	69.37	55.72	56.42	71.19	74.29	63.07	67.62
**TAG**	**91.28**	**83.04**	**73.47**	**72.38**	**87.17**	**78.36**	**85.02**	**81.53**	**91.72**	**70.65**	**60.53**	**58.63**	**72.52**	**76.06**	**79.28**	**72.77**

### Ablation study

We perform an ablation study for TAG and report the results in Table 7. The methods w/o curriculum and w/o node-level are TAG without the curriculum learning and the two-staged structure performing only graph-level contrastive learning, respectively. We also run TAG while fixing the proposed augmentations. black Since TAG needs both feature and structure augmentation algorithms to conduct two-staged contrastive learning, we evaluate the performance of pairs of algorithms. For example, the ‘Edit feature + Delete node’ runs TAG using ‘edit feature’ and ‘delete node’ algorithms for feature and structure modification, respectively.

**Table 7:** Ablation study for TAG. We report accuracies of graph classification using SVM and MLP classifiers. Bold, underlined, and Avg. texts denote the best, the second-best, and the average accuracy, respectively. The methods w/o curriculum and w/o node-level refer to TAG without the curriculum learning and the node-level contrastive learning, respectively. The fixed augmentation methods (Edit feature + Delete node, Edit feature + Delete edge, etc.) run TAG by using the same feature and structure augmentations for all graphs, while TAG randomly selects an augmentation for each graph. Note that TAG shows the best performance for all cases.

Method	Unsupervised Setting (SVM)								Unsupervised Setting (MLP)							
	MUT.	PROT.	NCI1	N109	DD	PTC	DBLP	Avg.	MUT.	PROT.	NCI1	N109	DD	PTC	DBLP	Avg.
w/o curriculum	90.07	78.14	65.99	64.66	80.36	70.08	78.06	75.33	91.18	67.58	54.88	55.15	69.73	70.77	76.68	69.42
w/o node-level	90.40	77.07	65.20	65.25	80.14	69.32	77.48	74.98	86.09	66.80	54.36	55.06	70.34	70.94	76.71	68.61
Edit feature + Delete node	90.70	80.75	67.25	65.06	81.09	**79.41**	80.54	77.81	92.70	69.90	58.08	56.91	**73.91**	75.10	77.39	71.76
Edit feature + Delete edge	90.54	81.11	66.61	64.41	80.25	79.04	83.14	77.87	87.55	69.96	59.55	58.78	68.60	76.58	76.99	71.14
Edit feature + Cut subgraph	90.82	80.85	66.13	63.54	80.08	66.30	78.58	75.19	92.47	**71.17**	55.16	56.58	67.63	74.94	77.33	70.72
Mix feature + Delete node	90.76	79.93	**74.70**	72.91	86.32	71.93	82.71	79.83	92.94	70.35	**60.58**	**60.16**	65.98	74.47	72.96	70.89
Mix feature + Delete edge	90.65	81.98	73.05	73.91	85.52	77.77	83.56	80.85	88.30	70.01	59.32	57.31	70.71	70.26	73.17	69.87
Mix feature + Cut subgraph	90.77	78.95	74.55	**74.82**	82.54	78.56	83.03	80.45	**93.10**	69.45	60.10	56.19	69.40	**77.14**	78.00	71.89
Add noise + Delete node	90.83	80.63	67.06	64.35	82.11	71.59	78.45	76.43	91.37	68.36	56.94	58.10	68.70	72.89	76.91	70.47
Add noise + Delete edge	90.36	81.05	65.44	64.63	80.75	71.90	83.30	76.78	91.62	69.49	58.86	55.92	70.76	76.81	77.30	71.54
Add noise + Cut subgraph	90.75	81.30	66.62	65.84	81.11	71.20	79.04	76.55	90.01	70.73	58.01	58.13	69.24	75.58	77.67	71.34
**TAG**	**91.28**	**83.04**	73.47	72.38	**87.17**	78.36	**85.02**	**81.53**	91.72	70.65	60.53	58.63	72.52	76.06	**79.28**	**72.77**

TAG with the curriculum learning improves the classification performance of SVM and MLP by 6.20% and 3.35% points on average, respectively, compared to that without the curriculum learning. Using both node-level and graph-level contrastive learning on TAG achieves 6.55% and 4.16% points higher average accuracy than using only graph-level contrastive learning on TAG in SVM and MLP classifiers, respectively. Experimental results of fixing the proposed augmentations show higher accuracies than the methods w/o curriculum and w/o node-level. The results prove that the proposed augmentation algorithms preserve the semantics well since the accuracies of the fixed augmentation methods are comparable to TAG. Furthermore, TAG achieves the best performance when it utilizes all the proposed augmentation algorithms. The results show that the proposed ideas, i.e., the two-staged framework, exploitation of curriculum learning, and the proposed augmentation algorithms for contrastive learning improve the accuracy of graph classification.

## Conclusion

We propose TAG, a two-staged contrastive curriculum learning model for graphs. We introduce two types of data augmentations for graphs and propose six model-agnostic augmentation algorithms that minimize information loss. TAG conducts contrastive curriculum learning in two stages. In the first stage, TAG gathers the relational information between nodes from an original graph and a feature-modified graph. In the second stage, the proposed method utilizes both feature-modified and structure-modified graphs to learn the similarity between them. We exploit curriculum learning to effectively train the model via carefully selected ordering of negative samples. We evaluate TAG by measuring the graph classification accuracy and running time. TAG shows the fastest running time and the best accuracy achieving up to 4.08% points and 4.76% points higher average accuracy than the second-best competitors in unsupervised and supervised settings, respectively. Future works include designing an accurate graph classification method for hypergraphs.
